# Potential for enhancing efficacy of screening colonoscopy by lowering starting ages and extending screening intervals: A modelling study for Germany

**DOI:** 10.1002/ijc.35322

**Published:** 2025-01-03

**Authors:** Dmitry Sergeev, Thomas Heisser, Michael Hoffmeister, Hermann Brenner

**Affiliations:** ^1^ Division of Clinical Epidemiology and Aging Research German Cancer Research Center (DKFZ) Heidelberg Germany; ^2^ Medical Faculty Heidelberg, Heidelberg University Heidelberg Germany; ^3^ German Cancer Consortium (DKTK), German Cancer Research Center (DKFZ) Heidelberg Germany

**Keywords:** colonoscopy, colorectal cancer, endoscopy, modelling, screening

## Abstract

Studies aimed to evaluate the expected impact of alternative screening strategies are essential for optimizing colorectal cancer (CRC) screening offers, but such studies are lacking in Germany, where two screening colonoscopies (CS) 10 years apart are offered for men from age 50 and women from age 55. Our aim was to explore whether and to what extent the efficacy of utilizing two CS could be enhanced by alternative starting ages and screening intervals. We modeled the expected numbers of CRC cases, CRC deaths, years of potential life lost (YPLL), and disability‐adjusted life years (DALYs) due to CRC in hypothetical cohorts of 100,000 men and women aged 45–85 using COSIMO, a validated Markov‐based multi‐state simulation model. Modeled strategies included combinations of starting ages (45/50/55/60) and CS (10/15/20 years). For men, CRC deaths could be slightly reduced by extending the interval to 15 years, with a second CS at 65. YPLL and DALYs would be reduced by decreasing starting age to 45 when combined with a 15‐year screening interval. For women, use of two CS at ages 50 and 65 would reduce all CRC burden parameters compared to the current earliest‐use offer at 55 and 65 years. Our results suggest that lowering the starting age of screening colonoscopy to 45 for men and 50 for women, combined with extending the CS screening interval to 15 years would have the potential to enable significant reductions in years of potential life lost, and disability‐adjusted life years compared to current screening offers in Germany.

## What's New?

Recent reductions in the age‐of‐onset of colorectal cancer (CRC), along with increasing CRC risk with age, warrant reevaluation of CRC screening guidelines. Here, using Markov‐based modeling, the authors explored possibilities for more efficient use of two screening colonoscopies currently offered in Germany's CRC screening program. Modeling shows that compared to current strategies, both lowering the screening start age and extending the interval between colonscopies to 15 years allows for significant reductions in CRC cases, deaths, years of potential life lost, and disability‐adjusted life years. Incorporating these adjustments into the current two‐screening offer could help maximize CRC screening benefits in Germany.

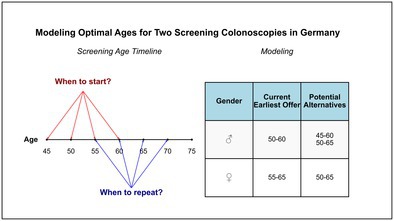

## 1 INTRODUCTION

Colorectal cancer (CRC) is a major cause of cancer morbidity and mortality worldwide. In Germany, like in many other high‐income countries, CRC is the second most common cause of cancer‐related deaths, accounting for approximately 24,000 deaths each year, even though age‐standardized incidence and mortality have declined by approximately 25%–35% since the introduction of screening colonoscopy (CS) in 2002.^1^

When first introduced, individuals covered by statutory health insurance in Germany were offered up to two screening CS 10 or more years apart, starting from age 55 for both men and women. In 2019, the starting age for men was reduced to 50 years. Hence, perfectly adherent men and women can make use of the CS offer by undergoing two screening CS, either at ages 50 and 60 (men) or at ages 55 and 65 (women) (Table S1). The limitation to two colonoscopies may reflect a practical choice amid considerations of capacities and costs. The 10‐year time interval between screening CS is in agreement with current recommendations by national and international expert panels.^2^ It implies, however, that perfectly adherent men and women would have no further CS offer beyond 60 or 65 years of age, even though most CRC cases in Germany occur above 70 years of age. However, recent studies demonstrating very low prevalences of CRC and its precursors 10 or more years after a negative first CS suggest that the time interval between screening CS might be extended beyond 10 years.^3^, ^4^, ^5^ Also, based on recent evidence of increasing CRC risks in younger age birth cohorts, the American Cancer Society (ACS)^6^ and the US Preventive Services Taskforce (USPSTF)^7^ lowered the recommended starting age to age 45 in 2018 and 2021, respectively.

The aim of this modelling study for Germany was to assess and compare the expected reduction of the CRC burden by different combinations of starting ages (including the currently not offered starting age of 45) and screening intervals for men and women, given a constraint of a maximum of two screening CS during their lifetime.

## 2 METHODS

### 2.1 Study concept

This study applied the Colorectal Cancer Multistate Simulation Model (COSIMO), a validated Markov‐based simulation tool, for modelling CRC outcomes expected with full adherence to various potential offers of screening CS in the eligible German population. Briefly, COSIMO simulates the natural history of CRC based on the incidence and progression of CRC precursor lesions developing into preclinical and then clinical cancer, and potential inference by interventions as outlined in detail elsewhere^8^ and visually summarized in Figure S1. We simulated data on hypothetical cohorts of 100,000 men and women followed from age 45 to age 85. Our goal was to identify the best possible starting age and screening interval combination while applying a restriction of a maximum of two lifetime CS, reflecting the current maximum offer in Germany, focusing on a range of outcome measures.

### 2.2 Model parameters

Model input parameters included the prevalences of non‐advanced adenomas, advanced adenomas, and preclinical CRC at age 45, as well as annual transition rates from no lesion to nonadvanced adenoma, from nonadvanced adenoma to advanced adenoma, from advanced adenoma to preclinical cancer, and from preclinical to clinically manifest cancer. From the pertinent prevalences by sex and 5‐year age groups observed among 3.6–4.3 million screening participants aged 55–59, 60–64, 65–69, 70–74, 75–79, and 80+ years in the German screening colonoscopy program between 2003 and 2013, age‐ and sex‐specific transition rates were derived as described in detail in several previous studies^9^, ^10^, ^11^ and are provided in Table S2. Given that screening CS was offered from age 55 on only in 2002–2013, estimated transition rates were available for age groups 55–59 years and older only, and the youngest age for which prevalences were available was age 55. Given the very high similarity of transition rates across age groups, we assumed the same transition rates for age groups 45–49 and 50–54 as those estimated for age group 55–59, and we determined “starting prevalences” of the various types of colorectal neoplasms at age 45 in an iterative process which yielded the best possible agreement in COSIMO‐based simulations with the prevalences observed at age 55. These starting prevalences at age 45 are provided in Table S3. We assumed diagnostic performance parameters for colonoscopy, with sensitivities of 75% for non‐advanced adenomas and 95% for advanced neoplasms‐based on evidence of polyp and adenoma miss rates determined by tandem colonoscopy, with ideal specificity (100%).^12^, ^13^ Since our population‐based model assigns global parameters to all subjects, it inherently accounts for differences in miss rates by polyp class.

COSIMO integrates natural history assumptions derived from data obtained from the German screening CS registry, recognized as the largest registry of its kind worldwide, adjusted for potential miss rates during colonoscopies.^14^, ^15^, ^16^, ^17^ Furthermore, annual CRC mortality rates after CRC diagnosis, contingent upon the method of CRC detection and year since diagnosis, were applied which have been derived from the long‐term follow‐up of a large cohort of CRC patients in Germany as previously described in detail^17^, ^18^ and are shown in Table S4. Additionally, data on general mortality rates and life expectancy were sourced from German population life tables^19^ and are displayed in Table S5. Details of the model structure and data sources are described in Appendix S1, including an overview of all parameters.

### 2.3 Modelled strategies

**FIGURE 1:**
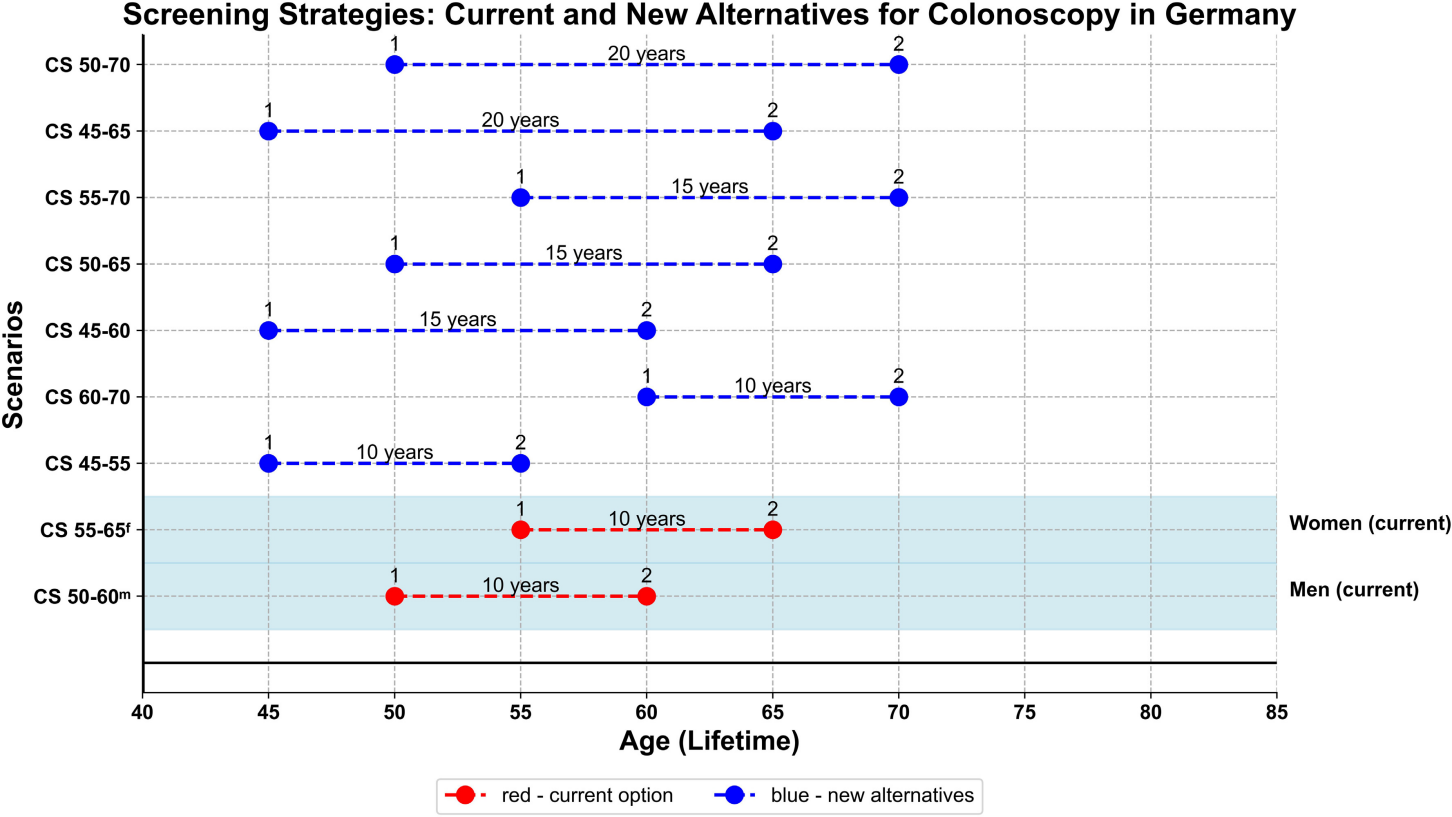
Colonoscopy screening scenarios assessed in the simulations.

We conducted simulations for nine different CRC screening scenarios, assuming full adherence to the offered screening interventions (Figure 1). Our objective was to evaluate screening effects under full adherence to screening, follow‐up, and surveillance. Simulations were performed separately for men and women. The simulation start age was set at 45 years for all models, even if the first CS was offered at a later age. For comparison, we simulated a scenario without screening. In all scenarios, individuals in whom no neoplastic polyp was detected at the first screening CS were assumed to have a second screening CS after the defined screening interval, unless they had a CRC diagnosis or died in the meantime. Subjects in whom adenomas were detected were assumed to have these adenomas removed and to undergo surveillance colonoscopies in 3‐year intervals (after detection of advanced adenomas) or 10 year intervals (after detection of nonadvanced adenomas).^20^ The age limit of 85 follows USPSTF guidelines, recommending individualized screening for ages 76–85 while screening after 85 is discouraged due to reduced benefits and increased risks.

### 2.4 Outcomes measures

For each scenario, we estimated the numbers of CRC cases and deaths, years of potential life lost (YPLL) and disability‐adjusted life years lost (DALY) due to CRC across the 40‐year period from 45 to 85 years. We also determined the corresponding absolute and relative reductions of these outcomes compared to existing screening recommendations for both genders. YPLL was calculated by multiplying the number of deaths at a given age by the residual life expectancy at that age. DALYs combined YPLL and years lived with disability (YLD), accounting for both mortality and the impact of disabling conditions. This outcome focused on both quality of life and premature death, relevant for public health planning. Disability weights, representing health losses from perfect health to death, were applied to assess the burden of colorectal cancer, considering symptoms and stage‐specific outcomes.^21^, ^22^

## 3 RESULTS

The model predicted 11,862 CRC cases and 5264 CRC deaths between ages 45 and 84 among 100,000 unscreened men (Table 1). For women, the corresponding numbers were 8899 and 3795. Substantially lower case numbers and deaths would be expected with any of the various screening strategies (Tables 2 and 3, Figures S2 and S3).

**TABLE 1:** Model‐based estimates of cumulative numbers of CRC cases, CRC deaths, YPLL and DALYs in the absence of screening.

CRC outcomes in the absence of screening				
Sex	Cases	Deaths	YPLL	DALYs
	*N*	*N*	*N*	*N*
Men	11,862	5264	69,173	126,843
Women	8899	3795	54,720	101,846

Abbreviations: CRC, colorectal cancer; DALYs, disability‐adjusted years of life lost; YPLL, years of potential life lost.

**TABLE 2:** Expected changes in CRC burden with deviation from current earliest screening colonoscopy offer for men in Germany (first and second screening colonoscopy at ages 50 and 60).

Screening interval [y]	Age at colonoscopy		CRC outcomes							
			CRC cases		CRC deaths		YPLL		DALYs	
	First	Second	*N*	Change^a^	*N*	Change^a^	*N*	Change^a^	*N*	Change^a^
	No screening		11,862	+294%	5264	+414%	69,173	+347%	126,843	+253%
10	45	55	3788	+26%	1384	+35%	15,562	±0%	38,331	+7%
	**50**	**60**	**3012**	**—**	**1024**	**—**	**15,487**	**—**	**35,928**	**—**
	55	65	3262	+8%	1101	+8%	20,428	+31%	47,143	+31%
	60	70	3945	+31%	1363	+33%	27,723	+79%	60,295	+68%
15	45	60	3210	+7%	1077	+5%	14,057	−9%	34,294	−5%
	50	65	3035	+1%	983	−4%	16,391	+6%	39,568	+10%
	55	70	3517	+17%	1168	+14%	22,020	+42%	49,230	+37%
20	45	65	3543	+18%	1156	+13%	17,033	+10%	42,801	+19%
	50	70	3600	+20%	1175	+15%	19,703	+27%	46,651	+30%

*Abbreviations*: CRC, colorectal cancer; DALYs, disability adjusted years of life lost; YPLL, years of potential life lost.

^a^ Compared to current earliest screening offer (numbers in grey‐shaded cells).

**TABLE 3:** Expected changes in CRC burden with deviation from current earliest screening colonoscopy offer for women in Germany (first and second screening colonoscopy at ages 55 and 65).

Screening interval [y]	Age at colonoscopy		CRC outcomes							
			CRC cases		CRC deaths		YPLL		DALYs	
	First	Secod	*N*	Change^a^	*N*	Change^a^	*N*	Change^a^	*N*	Change^a^
	No screening		8899	+291%	3795	+397%	54,720	+281%	101,846	+191%
10	45	55	3225	+42%	1190	+56%	14,073	−2%	36,454	+4%
	50	60	2413	+6%	839	+10%	12,365	−14%	30,514	−13%
	**55**	**65**	**2276**	**—**	**763**	**—**	**14,378**	**—**	**35,022**	**—**
	60	70	2500	+10%	822	+8%	18,624	+30%	41,670	+19%
15	45	60	2614	+15%	895	+17%	11,862	−17%	30,717	−12%
	50	65	2213	−3%	724	−5%	12,207	−15%	31,266	−11%
	55	70	2341	+3%	744	−2%	15,143	+5%	35,275	+1%
20	45	65	2598	+14%	850	+11%	13,207	−8%	34,946	±0%
	50	70	2508	+10%	784	+3%	14,337	±0%	34,382	−2%

*Abbreviations*: CRC, colorectal cancer; DALYs, disability adjusted years of life lost; YPLL, years of potential life lost.

^a^ Compared to current earliest screening offer (numbers in grey‐shaded cells).

**FIGURE 2:**
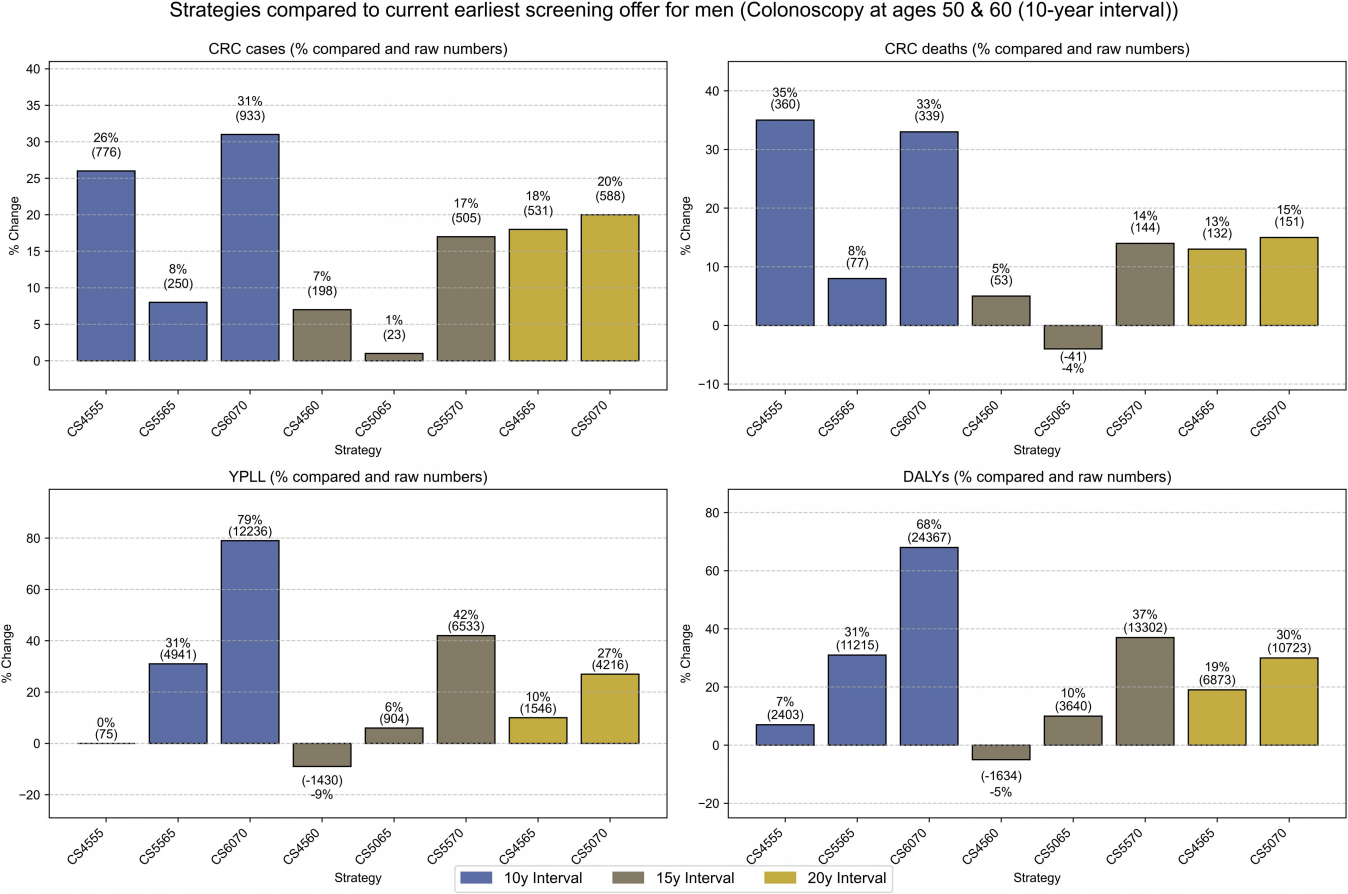
Comparative CRC outcomes in 100,000 men undergoing two screening colonoscopies at various ages and intervals: cases, deaths, YPLL, and DALYs compared to the earliest screening offer. CS, colonoscopy; CRC, colorectal cancer; DALYs, disability adjusted years of life lost; Y, year interval; YPLL, years of potential life lost; numbers indicate the ages at which the first and second colonoscopies are performed.

Among men (Table 2, Figure 2), screening at ages 50 and 60 could reduce CRC cases to 3012 and deaths to 1024, that is, to approximately one fourth and one fifth of the estimated numbers without screening. For all alternative offers of two screening CS 10 or 20 years apart, the expected numbers of both CRC cases and deaths, as well as YPLL and DALYs would be higher. However, with two screening CS at ages 45 and 60, both YPLL and DALYs would be expected to be lower (−9% and −5%, respectively), and with two screening CS at ages 50 and 65, the total number of CRC deaths would be expected to be slightly lower (−4%) than with the currently offered earliest screening CS use.

**FIGURE 3:**
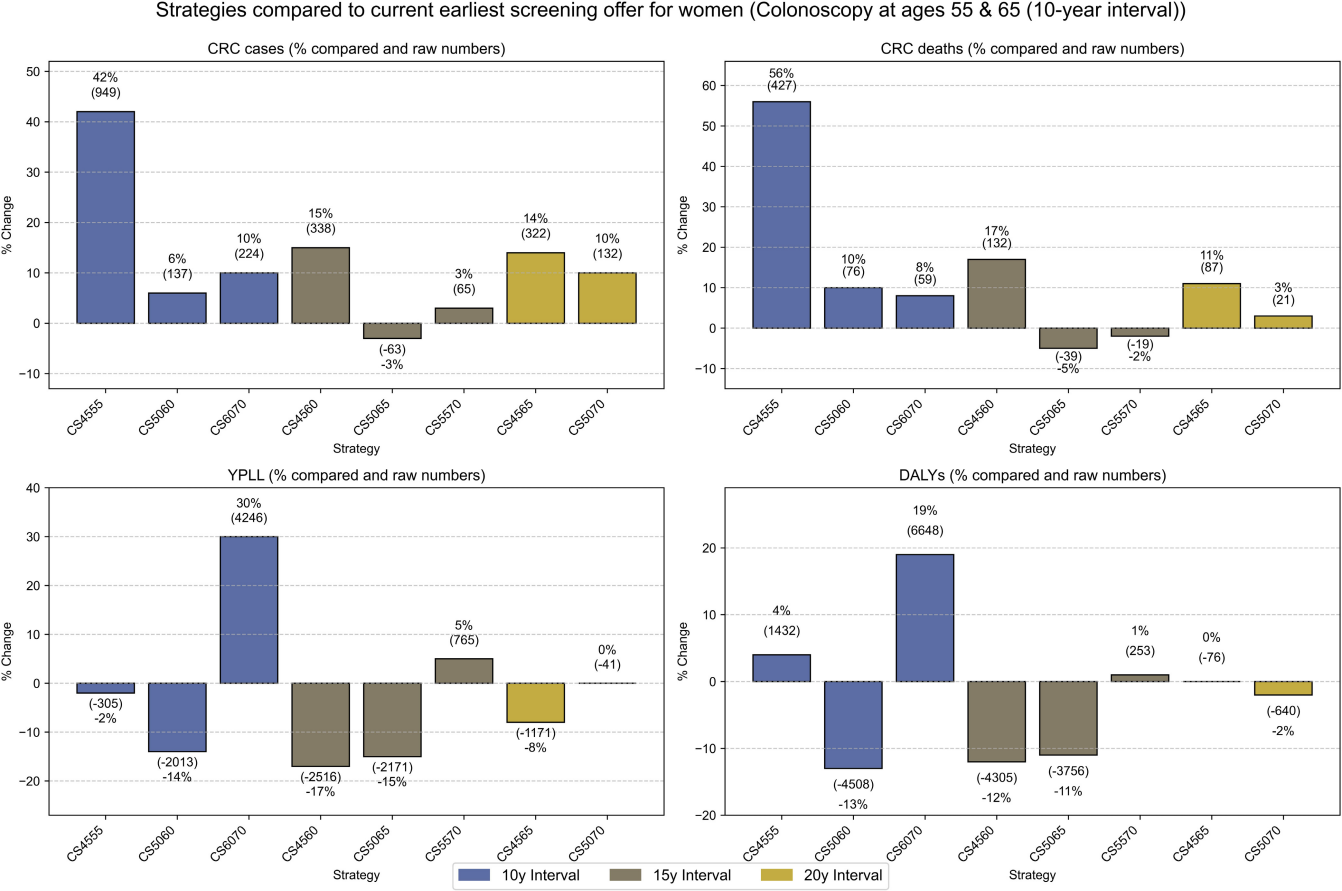
Comparative CRC outcomes in 100,000 women undergoing two screening colonoscopies at various ages and intervals: cases, deaths, YPLL, and DALYs compared to the earliest screening offer. CS, colonoscopy; CRC, colorectal cancer; DALYs, disability adjusted years of life lost; Y, year interval; YPLL, years of potential life lost; numbers indicate the ages at which the first and second colonoscopies are performed.

Among women (Table 3, Figure 3), the numbers of CRC cases and CRC deaths could be reduced to 2276 and 763 with earliest use of the current screening offers, that is, at ages 55 and 65. These numbers are again approximately one fourth and one fifth of the estimated numbers without screening. However, even stronger reductions in the various outcome parameters could be achieved by alternative screening strategies. Although this applied to different outcomes in different screening strategies, a stronger impact than with the current earliest use of the screening offers could be achieved with respect to all four outcome parameters with the use of two screening CS at ages 50 and 65. Superiority of this screening strategy was most pronounced with respect to YPLL (−15%) and DALYs (−11%).

## 4 DISCUSSION

In this study, we assessed and compared the reduction of the CRC burden that may be achieved for men and women in Germany, assuming full adherence to the offer of two screening CS at varying starting ages and screening intervals. Compared to no screening, all screening strategies would be expected to result in prevention of the majority of CRC cases and deaths, as well as YPLL and YDL, for both men and women. However, some potential to enhance efficacy over the current screening offers was identified. For men, the number of CRC deaths could be slightly reduced by using the second CS at age 65 rather than 60, thereby extending the screening interval to 15 years. However, to reduce rather than increase YPLL and DALYs, the starting age would have to be reduced to 45 years. Even though this would go along with a slightly increased total number of CRC deaths, the somewhat stronger reduction in YPLL might still favour such a “45/60 year sequence” for men. Among women, utilizing two CS at ages 50 and 65 would be expected to reduce all parameters of the CRC burden compared to the current “earliest‐use offer” at 55 and 65 years.

### 4.1 Findings in context

CRC screening guidelines and offers vary substantially across countries, both in terms of types of screening exams and starting ages and intervals of screening.^23^, ^24^ Although the most widely recommended, used and offered CRC screening tests are fecal occult blood tests, screening CS is a key pillar of CRC screening in several countries including Germany and the US. While the USPSTF and ACS recommend screening every 10 years from 45 to 75, for both genders,^6^, ^7^ a maximum of two screening CS starting from age 50 (men) or 55 (women) is offered in Germany.

Microsimulations for the US suggested that starting CRC screening at age 45 reduces CRC incidence and mortality and increases life expectancy compared to starting at age 50.^25^, ^26^ However, these microsimulations assumed that screening CS could be used more than twice every 10 years up to age 75, reflecting common practice in the US. In a previous simulation study for Germany by Chen et al., the optimal screening age for a single CS in Germany was estimated to be 54 for men and 56 for women, while for multiple screenings, an optimal starting age around 50 or younger was suggested for both genders.^18^ However, this study had not considered potential prolongation of screening intervals as suggested by several recent studies,^3^, ^4^, ^5^ and assessed outcomes had been restricted to CRC deaths and YPLL. Another previous modelling study for Germany demonstrated the incremental benefits of offering three rather than two screening CS in Germany,^27^ but again had not explored benefits that might be achieved with more limited resources and capacities that would allow only two screening CS over lifetime. Since the demand for screening CS, in contrast to screening CS capacities, is expected to strongly increase in the decades to come due to demographic aging in many countries, efforts to optimize use of restricted resources are of paramount importance.^28^

Whether or not or to what extent CRC screening offers should be gender‐specific is subject to ongoing debate. The rationale for gender‐specific screening recommendations is major differences in age‐specific CRC incidence and life expectancy between men and women. In most countries including Germany, age‐specific CRC incidence is substantially lower among women than among men, with women reaching comparable age‐specific CRC incidence rates at approximately 5 years higher ages than men.^29^ At the same time, life expectancy is substantially longer (by approximately 5 years) among women than among men. These patterns suggest that a difference in screening ages of approximately 5 years between men and women might reflect these epidemiological and demographic differences, which is in line with our results of potentially optimized screening CS offers at 45 and 60 years for men and 50 and 65 years for women. Earlier detection may catch cancer at more treatable stages, particularly in younger patients, potentially reducing both the incidence of advanced cases and the risk of premature death. Lowering the screening age could lead to fewer CRC cases and deaths—both of which are equally important in reducing the overall disease burden. However, cases and deaths alone do not fully capture the impact. YPLL, which measures the years of life lost due to premature death, emphasizing the additional burden that early CRC‐related deaths place on younger patients. DALYs, which include both YPLL and the years lived with disability, provide a more comprehensive view by assessing not only mortality but also the quality of life impacted by CRC, which is closely related to incidence.

On first view, our findings of very strong reduction of CRC incidence and mortality of all simulated screening approaches seem to be in conflict with much weaker effect estimates of screening CS suggested by recently reported preliminary results of the first randomized trial on long‐term effects of screening CS, the NordICC trial.^30^ However, apart from the low adherence rate to the screening offer (42%) in this trial, the so far very limited follow‐up time, during which results on CRC incidence to a large extent reflect early detected prevalent rather than prevented CRC cases, may explain the apparent inconsistencies.^31^, ^32^ The modelled strong reductions in CRC incidence and mortality compared with no screening are in line with a large body of evidence from observational studies and real‐life evidence of strong declines in CRC incidence and mortality that were selectively seen in screening ages in Germany and the US even with far from perfect screening adherence.^33^, ^34^

It is important to note that all of our analyses pertain to scenarios of full adherence to the screening offers. Under real life conditions, reduction of the burden of CRC due to screening will be substantially smaller due to limited adherence. Differences in adherence rates according to CS screening starting ages or intervals might result in somewhat different patterns regarding preferred screening strategies. For example, if adherence rates were lower for CS screening offered at younger ages, this could diminish potential benefits of lowering the starting age. Given the uncertainties on variation of adherence rates according to starting age and screening intervals we abstained from including them in our models. However, potential changes in adherence rates need to be carefully monitored when screening offers are changed.

For similar reasons, our modelling did not also include the alternative offer of screening by fecal occult blood tests for people not using screening colonoscopy in Germany. While overall use rates of fecal tests are low, it is uncertain to what extent they would vary in case of alternative starting ages or intervals of CS screening offers.

### 4.2 Strengths and limitations

Strengths of our analyses include use of a validated multistate transition model, for which sex‐ and age‐specific transition rates have been carefully derived from Germany's national screening CS registry, the largest of its kind globally. Modeled outcome parameters included not only CRC cases and deaths, but also YPLL and DALYs. However, our analyses also have a number of limitations which need to be carefully kept in mind when interpreting our results. First, transition rates for age groups 45–49 and 50–54 could not be directly derived from the German national screening CS registry but were assumed to be the same as those for age group 55–59 for both sexes in our analyses. Given that sex‐specific transition rates were rather constant across older age groups, this assumption appears well justified. Second, adenoma miss rates could not be directly derived from the data of the German national screening CS registry, but had to be based on meta‐analyses from studies conducted in different countries in our modelling. Third, our analyses were restricted to scenarios with a maximum of two screening CS and focused on comparisons with the earliest use of current screening CS offers in Germany. While this focus was motivated and justified to best inform potential further development of the German screening CS program, different scenarios may be more relevant in other settings. However, different screening strategies of interest in other settings or countries could equally be performed by COSIMO, and we encourage the use of this open‐source, transparent model for such applications.

### 4.3 Conclusion

In this study, we explored the impact of various CRC screening strategies in Germany within the constraints of two CS in lifetime offered by the German screening colonoscopy program. Results revealed robust protection against CRC development and mortality across age groups and intervals, emphasizing the overall efficacy of screening. Nevertheless, this study suggests that, with respect to YPLL and DALYs, lowering starting ages of screening colonoscopy, combined with extending the interval to 15 years could maximize the benefits of the current “two screening CS offer” in Germany. In addition, efforts to enhance use of screening offers, in particular among high‐risk groups, and enhanced efforts of primary prevention will be crucial to limit the increasing CRC burden that is otherwise to be expected by demographic changes in the decades to come.^28^

## AUTHOR CONTRIBUTIONS

**Dmitry Sergeev:** Conceptualization; writing – original draft; investigation; methodology; visualization; formal analysis; data curation. **Thomas Heisser:** Writing – review and editing; validation; formal analysis; software; data curation. **Michael Hoffmeister:** Writing – review and editing. **Hermann Brenner:** Conceptualization; investigation; funding acquisition; methodology; writing – review and editing; project administration; supervision; resources; validation.

## FUNDING INFORMATION

This study was supported by grants from The German Cancer Aid (No. 70114735) and the German Federal Ministry of Education and Research (No. 01KD2104A). The funding agreements ensured the authors' independence in designing the study, interpreting the data, writing, and publishing the report.

## CONFLICT OF INTEREST STATEMENT

The authors declare no conflicts of interest.

## Supporting information


**Appendix S1:** Supporting information.

## Data Availability

All analyses relevant to the study are included in the article or uploaded as supplementary information. The model source code is available from https://www.dkfz.de/en/klinepi/download/index.html. Further information is available from the corresponding author upon request.
