# Supplementary material for: Potential for enhancing efficacy of screening colonoscopy by lowering starting ages and extending screening intervals: A modelling study for Germany
Source: Int J Cancer. 2025 Jan 3;156(12):2303–10. doi: 10.1002/ijc.35322 (PMC12008824; doi:10.1002/ijc.35322)
Supplement: Supplementary file 1 — Appendix S1: Supporting information. [file IJC-156-2303-s001.pdf]

SUPPLEMENTARY MATERIAL

Potential for enhancing efficacy of screening colonoscopy by lowering starting ages and extending screening intervals: A modelling study for Germany.

Dmitry Sergeev, Thomas Heisser, Michael Hoffmeister, Hermann Brenner

Table of Contents:

Supplementary Appendix. COSIMO Model Documentation..........2
Supplementary Tables and Figures..........7
Supplementary References..........16

Supplementary Appendix. COSIMO Model Documentation

Conceptual model structure

Our Markov-based Colorectal Cancer Multistate Simulation Model (COSIMO) simulates the natural history of CRC based on the process of precursor lesions (non-advanced and advanced adenomas) developing into preclinical (asymptomatic) and then clinical (symptomatic) cancer. The simulation is performed on a hypothetical previously unscreened German population, with the number of simulated subjects and their corresponding baseline age (minimum 45 years) being variables to be chosen prior to model start. COSIMO can principally be used for simulating any population, provided updated or appropriately adjusted input parameters.

At start of the simulation, certain proportions of no neoplasm, non-advanced adenoma, advanced adenoma and preclinical CRC are assigned to the hypothetical population. The simulation runs up to a predefined number of cycles of each one year. Each year, people at each state have a certain probability (transition rate) to progress to the next state. Subjects with CRC may die from the disease, and at each state people may experience non-CRC death, reflecting the general background mortality from other causes.

Screening can alter the progression between states. People in whom adenomas are detected are moved backward to the state of no neoplasm, assuming removal of their adenoma at colonoscopy. Subjects will then continue to have the probabilities to progress to the next states as those without findings at screening. We assume that, although these people are under a higher risk of developing adenomas or cancers than the general population [1], the excess risk will be effectively compensated through the protection provided by surveillance colonoscopies [2, 3]. Preclinical CRC detected at screening are moved forward to the state of diagnosed cancer. After each cycle in which a colonoscopy screening test is applied, the model categorizes the simulated population based on the screening outcome, enabling the modeling of different trajectories. In these scenarios, individuals are eligible for the next screening round only if they had a negative result in the previous round. Subjects among whom non-advanced adenomas were detected are assumed to undergo surveillance colonoscopies at intervals of 10 years up to a predefined end age of 75 years. In case an advanced adenoma was detected, either at the primary screening test or at a surveillance colonoscopy, subjects are assumed to undergo periodic surveillance colonoscopies at three-yearly intervals up a predefined end age of 85.

The model source code, developed in the statistical software R (version 4.2.2 (2022-10-31)), is available for download from our website [4].

Model parameters

Starting prevalences and transition rates

An overview of key model parameters is given in **Supplementary Table 2**.

Data source

The data basis of our analyses on model starting prevalences and transition rates was the nationwide screening colonoscopy registry run by the Central Research Institute of Ambulatory Health Care in Germany. The registry, which was built up along with the introduction of the screening colonoscopy offer in the year 2002, is a repository of all screening colonoscopies conducted in Germany. Reporting has been virtually complete, as it is a prerequisite for physicians' reimbursement by the health insurance funds. The registry includes only primary screening examinations (i.e., colonoscopies conducted for surveillance, work-up of symptoms or other screening tests are not included). Items reported include, besides basic sociodemographic variables, findings at colonoscopy, including number, size and histological characteristics of polyps. In case of multiple neoplasms, only the most advanced one (non-advanced adenoma, advanced adenoma, or cancer) is recorded. Advanced adenomas are defined as at least 1 adenoma ≥ 1 cm or at least 1 adenoma with villous components or high-grade dysplasia.

The reporting for the screening colonoscopy registry does not differentiate by the class of lesion. Thus, the herein used term 'adenoma' refers to conventional or serrated adenomas (polyps) alike. While we preferred to refer to our model as being based on the adenoma-carcinoma pathway in previous publications [5-9] for the sake of simplicity and comprehensibility (as the grand majority of CRCs develops through this well-established pathway of cancer development [10, 11]), in fact COSIMO's defining parameters were derived using polyp/adenoma prevalences as detected and reported at screening colonoscopy, regardless of their underlying mechanism or pathway of development. Therefore, it will be more precise to refer to the model as being based on the 'natural history of CRC', without restrictions on underlying CRC development pathways.

Starting prevalence

The proportions of no neoplasm, non-advanced adenoma, advanced adenoma and preclinical CRC at the beginning of simulation were calculated based on the data from 344,658 participants of the German screening colonoscopy program who had their first screening colonoscopy during 2003–2012 at the age of 55 years [7]. To take into account that a certain proportion of neoplasms needs to be assumed to have been missed at colonoscopy screening, in particular for serrated or flat polyps [12, 13], we re-calculated the previously reported

prevalences [7], assuming representative miss rates of 25% for non-advanced adenomas and 5% for advanced neoplasms (advanced adenomas and preclinical cancers).

This was used as the best estimate for simulations starting with a 50-year-old population, which seems reasonable as selected regional programs which offer screening colonoscopy from age 50 on found similar prevalences of adenomas in age groups 50-54 and 55-59 [14].

Prediction of prevalence at age 45 to inform modeling parameters

To estimate the starting prevalences at age 45, we utilized a backward simulation approach based on our Markov model of colorectal cancer's natural history. Since the model was originally calibrated with initial states at age 50, we needed to adjust these states to reflect the expected lower prevalences at age 45.Directly simulating backward in time is not feasible, so we modified the initial state probabilities at age 50. We applied a discount factor to the probabilities of being in preclinical disease states—specifically, non-advanced adenomas, advanced adenomas, and preclinical cancers. This reduction accounts for the lower prevalence of these conditions at a younger age. With this adjusted initial state vector representing age 45, we ran the Markov model forward from age 45 to 100, simulating disease progression over time for a large cohort (100,000 simulations). This process generated cumulative prevalences for each age, from which we extracted the proportions at age 45. These proportions represent the backward simulated starting prevalences at age 45, grounded in the natural history of the disease and accounting for prior screening effects. (**Supplementary Table 3**)

Transition rates

Transition rates between states were estimated based on data from the nationwide screening colonoscopy registry by several separate birth cohort and mean sojourn time analysis. Details on the principles of these methods have been described previously [15-17]. Briefly, sex- and age-specific annual incidence and transition rates were estimated from sex- and age-specific prevalences of adenomas among 3.6 – 4.3 million screening participants from the same birth cohorts in 2003–2011 (2003-2009) and 2004–2012 (2004 – 2010). The analysis on mean sojourn time of preclinical cancers additionally incorporated registry-reported colorectal cancer incidence and participation rates in screening colonoscopy from 2003-2006.

Similar as for the starting prevalences, as colonoscopy was shown to be less effective in detecting serrated lesions (and as the true proportions of missed conventional adenomas and serrated lesions in the registry-reported prevalences is unknown), we re-calculated previously reported transition rates [15-17] to adjust for representative colonoscopy miss rates [12, 13]. This adjustment resulted in slightly higher overall prevalences of adenomas, and therefore (when compared to previously reported rates) in slightly higher transition rates of incidence adenomas, as well as slightly lower transition rates from non-advanced to advanced adenomas

and from adenomas to cancer. Furthermore, to adjust for uncertainties resulting from the cycle length of one year used in COSIMO, we updated the model to allow for small proportion of subjects with very rapidly progressing lesions with limited potential for early detection and associated worse prognosis. Age- and sex-specific annual transition rates between the states were estimated for age groups from 55-79 years in steps of 5 years. Estimates for age 45-54 and ≥ 80 (or ≥ 85) were assumed to be the same as those for age group 55-59 and 75-79 (or 80-84), respectively.

Confidence intervals for both starting prevalences and transition rates were derived by bootstrap analysis with resampling within sex- and age-specific subgroups. Ninety-five percent confidence intervals were determined as the 2.5th and 97.5th percentile of transition rate estimates obtained in 1,000 runs.

Mortality rates

Mortality rates for patients whose cancer was detected by screening or by symptoms were estimated in previous analyses [8, 9]. We combined data on the proportion of screening-detected cases among all CRC cases in Germany during 2003-2012 in people aged 55-79 years [5, 18] with the overall CRC-specific mortality rates by year after diagnosis in Germany in 2011-2012 [18]. We then used hazard ratios for patients detected by screening versus symptoms as obtained from a German population-based case-control study on CRC screening with long-term mortality follow-up of CRC patients [9, 19] to estimate CRC-specific mortality rates by mode of detection (**Supplementary Table 4**). Sex- and age-specific general mortality rates and average life expectancy of the population were extracted from German population life tables 2010/2012 (**Supplementary Table 5)** [20].

Model validation

COSIMO has been validated for the German screening-eligible population. Details on the model validation process can be found in the literature [21]. Briefly, we pursued a three-fold approach using the best available evidence from epidemiological data sources in Germany. We compared model-derived cumulative incidence and prevalences of colorectal neoplasms to (a) results from KolosSal, a study in German screening colonoscopy participants, (b) registry-based estimates of CRC incidence in Germany, and (c) outcome patterns of randomized sigmoidoscopy screening studies. This approach enabled us to scrutinize the model's natural history component (Parts a and b) as well as the modeled effect of screening colonoscopy (Parts b and c) at the same time.

We found that (a) more than 90% of observed prevalences in the KolosSal study were within the 95% confidence intervals of the model-predicted neoplasm prevalences; (b) the 15-year

cumulative CRC incidences estimated by simulations for the German population deviated by 0.0% to 0.2% units in men and 0.0% to 0.3% units in women when compared to corresponding registry-derived estimates; and (c) the time course of cumulative CRC incidence and mortality in the modeled intervention group and control group closely resembles the time course reported from sigmoidoscopy screening trials. Overall, COSIMO adequately predicted colorectal neoplasm prevalences and incidences in a German population for up to 25 years, with estimated patterns of the effect of screening colonoscopy resembling those seen in registry data and real-world studies.

Summary

COSIMO is a Markov-based tool designed to simulate the natural history of CRC. The model operates on a hypothetical previously unscreened population, typically beginning at age 45. It tracks the progression of CRC from precursor lesions (non-advanced and advanced adenomas) through to preclinical (asymptomatic) and clinical (symptomatic) cancer. At the start of the simulation, individuals are assigned to states of no neoplasm, non-advanced adenoma, advanced adenoma, or preclinical CRC. The simulation proceeds in annual cycles, with each individual having a certain probability (transition rate) to move to the next state or die from CRC or other causes. Screening interventions can alter these transition rates, with adenomas detected and removed during colonoscopy leading to a reversion to the no neoplasm state. This model assumes that individuals with adenomas are at a higher risk of recurrence but are effectively managed through surveillance colonoscopies. After each cycle of colonoscopy screening, the model classifies the population based on the results, allowing different outcome trajectories. Only those with a negative result in the previous round proceed to the next screening.

COSIMO implemented in R version 4.2.2 (2022-10-31), has been validated against various epidemiological data sources, demonstrating its accuracy in predicting CRC prevalence, incidence, and the impact of screening strategies.

Supplementary Tables and Figures

Supplementary Table 1. *Screening offer in Germany since April 2019*[b]

Screening Test	Offers for Men	Offers for Women
**Colonoscopy**	Start of eligibility: age 50	Start of eligibility: age 55
	Up to two screening colonoscopies 10 years apart; 2nd screening colonoscopy only if 1st one was used before age 65	Up to two screening colonoscopies 10 years apart; 2nd screening colonoscopy only if 1st one was used before age 65
**Fecal immunochemical test (FIT)**[c]	Annually from age 50 to 54 if no screening colonoscopy is used	Annually from age 50 to 54
	Biennially from age 55 onwards if no screening colonoscopy is used	Biennially from age 55 onwards if no screening colonoscopy is used

[a] *The information was from reference [22].*
[b]*From October 2002 to March 2019, screening colonoscopy was offered from aged 55 on for both sexes. In April 2019, the eligibility age was lowered for men to age 50, while the offer for women remained unchanged.*
[c] *Following a positive FIT, diagnostic colonoscopy is warranted. The traditionally used guaiac-based stool tests were replaced by modern FITs in 2017.*

Supplementary Table 2. Overview of model parameters: sex- and age-specific annual transition rates between states[1]

		Annual transition rates % (95% confidence interval)				
**Sex**	**Age**	**No neoplasm to non-advanced adenoma**	**Non-advanced adenoma to advanced adenoma**	**Advanced adenoma to preclinical colorectal cancer**	**Preclinical colorectal cancer to clinical colorectal cancer**	**Preclinical colorectal cancer to colorectal cancer death**
Men	55-59[2]	3.1 (2.9 – 3.4)	3.3 (2.8 – 3.9)	2.6 (2.2 – 3.1)	15.5 (14.9 – 16.6)	1.5 (1.4 – 1.6)
	60-64	3.1 (2.8 – 3.4)	3.2 (2.6 – 3.7)	3.1 (2.6 – 3.4)	16.4 (15.7 – 17.4)	1.6 (1.6 – 1.7)
	65-69	3.2 (2.9 – 3.4)	3.2 (2.6 – 3.7)	3.8 (3.4 – 4.3)	18.2 (17.4 – 19.1)	1.8 (1.7 – 1.9)
	70-74	2.9 (2.6 – 3.3)	3.3 (2.6 – 4.0)	5.1 (4.5 – 5.8)	17.6 (16.8 – 18.5)	1.7 (1.7 – 1.8)
	75-79	2.3 (1.8 – 2.9)	3.0 (1.9 – 4.2)	5.2 (4.2 – 6.2)	17.3 (16.3 – 18.3)	1.7 (1.6 – 1.8)
	80+	2.3 (1.8 – 2.9)	3.0 (1.9 – 4.2)	5.2 (4.2 – 6.2)	15.7 (14.5 – 17.1)	1.6 (1.4 –1.7)
Women	55-59[2]	1.8 (1.7 – 2.0)	3.2 (2.6 – 3.8)	2.5 (2.0 – 2.9)	18.2 (16.8 – 19.7)	1.9 (1.8 – 2.1)
	60-64	2.0 (1.8 – 2.2)	2.9 (2.2 – 3.4)	2.7 (2.2 – 3.2)	19.1 (17.8 – 20.3)	2.0 (1.9 – 2.1)
	65-69	2.1 (1.9 – 2.3)	2.9 (2.3 – 3.5)	3.8 (3.3 – 4.3)	18.7 (17.7 – 19.7)	2.0 (1.9 – 2.1)
	70-74	2.0 (1.7 – 2.2)	3.8 (3.0 – 4.6)	5.0 (4.2 – 5.7)	17.8 (16.8 – 18.9)	1.9 (1.8 – 2.0)
	75-79	1.6 (1.1 – 2.0)	3.0 (1.7 – 4.4)	5.6 (4.4 – 6.8)	16.5 (15.5 – 17.7)	1.7 (1.6 – 1.9)
	80+	1.6 (1.1 – 2.0)	3.0 (1.7 – 4.4)	5.6 (4.4 – 6.8)	14.9 (13.9 – 16.1)	1.6 (1.4 – 1.7)

[1] Estimates extracted and recalculated from references [15-17]

[2] Transition rates for the 55-59 age group were applied to the 45-49 and 50-54 age groups.

Supplementary Table 3. Model-derived proportions of men and women with no neoplasm, non-advanced adenoma, advanced adenoma and preclinical CRC at age 45[1]

Sex	Most advanced finding [%]			
	No neoplasm	Non-advanced adenoma	Advanced adenoma	Preclinical colorectal cancer
**Men**	84.23	11.95	3.43	0.37
**Women**	90.60	7.20	1.86	0.20

[1] Estimates based on an iterative backward-simulation

Supplementary Table 4. Annual CRC-specific mortality rates of CRC patients by mode of cancer detection[1]

Year after diagnosis	Annual CRC-specific mortality rates (%)			
	Screening colonoscopy–detected cases		Symptom-detected cases	
	Men	Women	Men	Women
1	4.6	3.7	19.7	20.6
2	2.2	1.9	9.3	10.7
3	2.1	1.3	8.8	7.4
4	1.5	0.9	6.3	4.8
5	1.2	0.6	5.0	3.3
6	0.8	0.3	3.5	1.7
7	0.4	0.3	1.8	1.8
8	0.4	0.3	1.9	1.8
9	0.4	0.0	1.9	0.0
10	0.0	0.0	0.0	0.0

[1] Estimates extracted from references [8, 9]
CRC, Colorectal cancer.

Supplementary Table 5. Sex- and age-specific general mortality rates

Age	General mortality rates from age to age +1 (%)[1] Men	Women
45	0.2	0.1
46	0.2	0.1
47	0.3	0.1
48	0.3	0.2
49	0.3	0.2
50	0.4	0.2
51	0.4	0.2
52	0.5	0.3
53	0.6	0.3
54	0.6	0.3
55	0.7	0.4
56	0.7	0.4
57	0.8	0.4
58	0.9	0.4
59	1.0	0.5
60	1.0	0.5
61	1.1	0.6
62	1.2	0.6
63	1.3	0.7
64	1.4	0.7
65	1.5	0.8
66	1.7	0.9
67	1.8	0.9
68	1.9	1.0
69	2.1	1.1
70	2.2	1.2
71	2.4	1.3
72	2.7	1.4
73	3.0	1.6
74	3.3	1.8
75	3.7	2.1
76	4.1	2.4
77	4.6	2.7
78	5.2	3.1

Continued on next page

Supplementary Table 5. *Sex- and age-specific general mortality rates (continued)*

	General mortality rates from age to age +1 (%)[1]	
Age	Men	Women
80	6.5	4.1
81	7.2	4.7
82	8.0	5.4
83	8.9	6.2
84	9.9	7.1

[1]Estimates were extracted from German population life tables 2010/2012 (reference [20]).

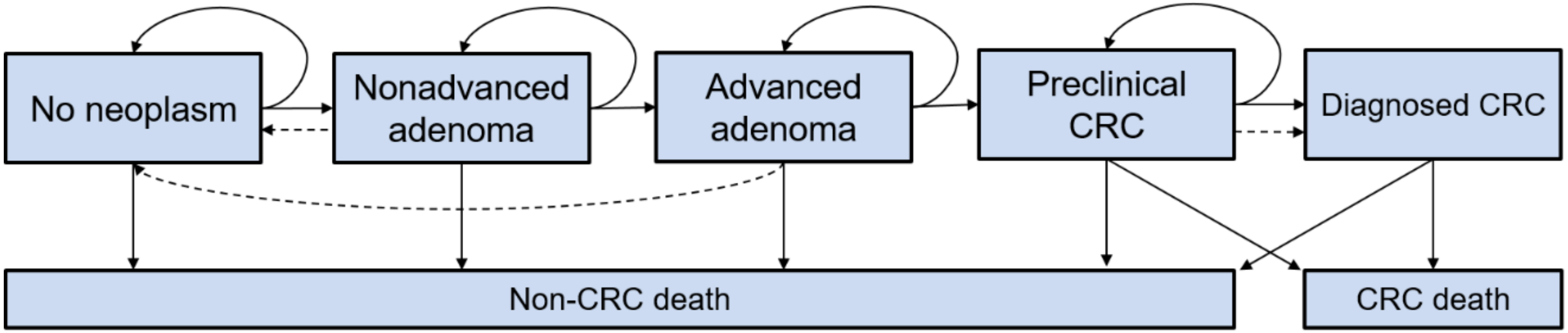

Supplementary Figure 1. ***Schematic illustration of the Colorectal Cancer Multistate Simulation Model (COSIMO)***

Solid lines represent the progression of colorectal disease through the adenoma-carcinoma sequence in the absence of screening; dashed lines show the movement between states because of the detection and removal of adenomas and the detection of asymptomatic CRC at screening.

CRC: Colorectal cancer.

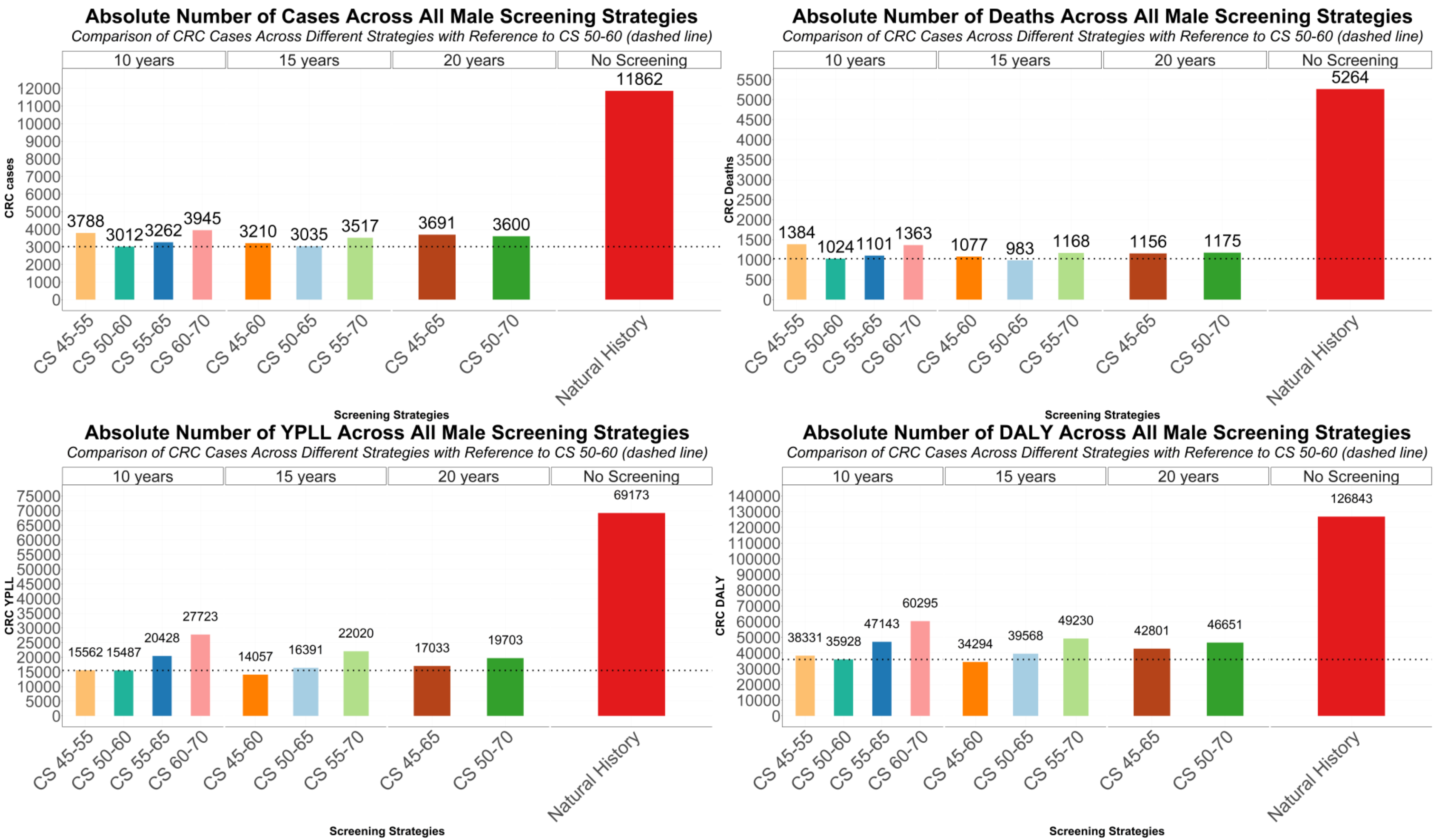

Supplementary Figure 2. Comparison of CRC Outcomes Across Male Screening Strategies: CRC Cases, Deaths, PYLL, and DALYs with Reference to CS 55-65 and Natural History(No Screening).

CRC: Colorectal cancer ; DALYs, disability adjusted years of life lost; YPLL, years of potential life lost

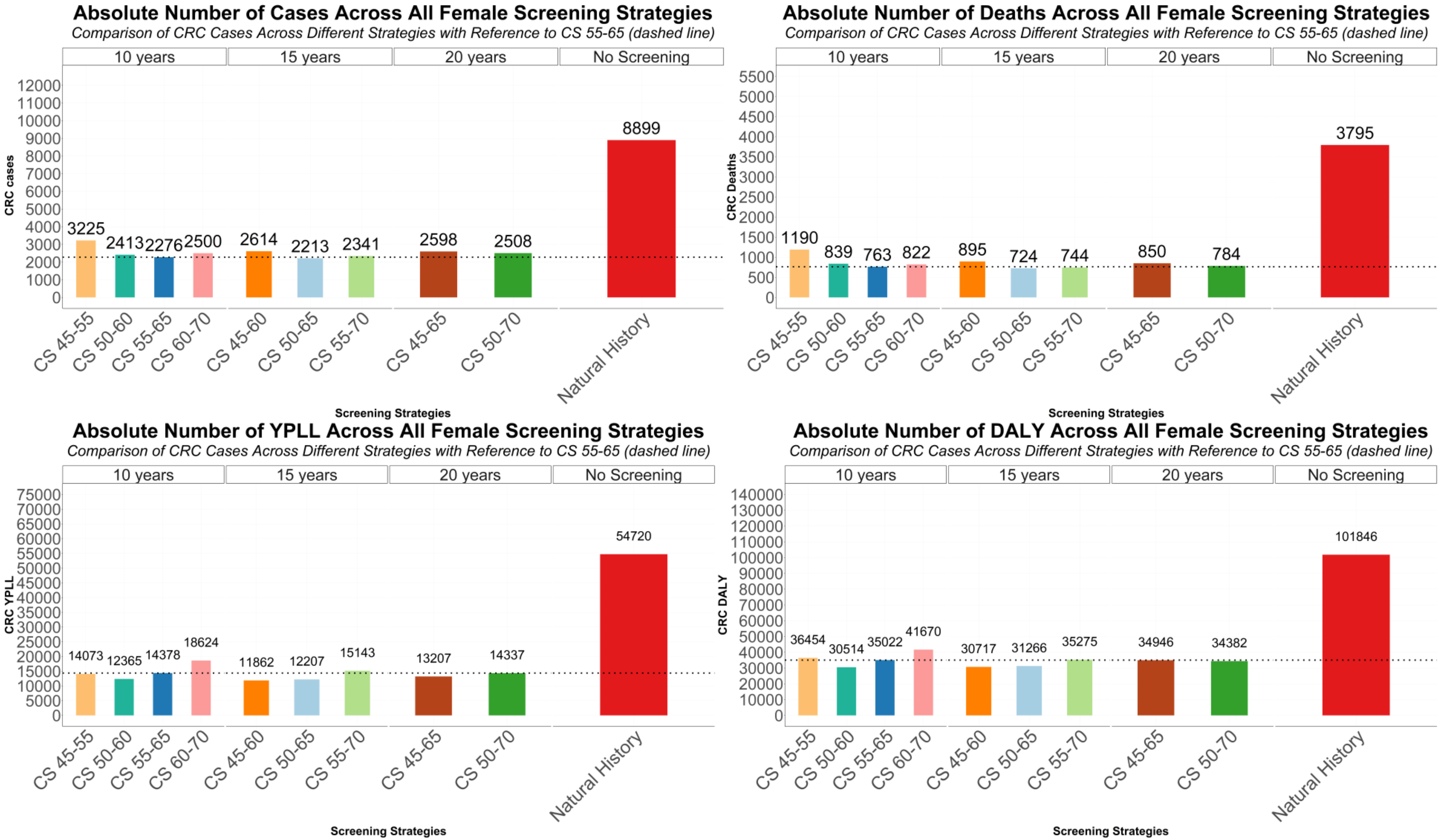

Supplementary Figure 3. Comparison of CRC Outcomes Across Female Screening Strategies: CRC Cases, Deaths, PYLL, and DALYs with Reference to CS 55-65 and Natural History(No Screening).

CRC: Colorectal cancer ; DALYs, disability adjusted years of life lost; YPLL, years of potential life lost

Supplementary References

[1] Cottet V, Jooste V, Fournel I, Bouvier A-M, Faivre J, Bonithon-Kopp C. Long-term risk of colorectal cancer after adenoma removal: a population-based cohort study. Gut. 2012;61:1180-6.

[2] Lieberman DA, Rex DK, Winawer SJ, Giardiello FM, Johnson DA, Levin TR. Guidelines for colonoscopy surveillance after screening and polypectomy: a consensus update by the US Multi-Society Task Force on Colorectal Cancer. Gastroenterology. 2012;143:844-57.

[3] Hassan C, Quintero E, Dumonceau J-M, Regula J, Brandão C, Chaussade S, et al. Post-polypectomy colonoscopy surveillance: European Society of Gastrointestinal Endoscopy (ESGE) Guideline. Endoscopy. 2013;45:842-51.

[4] German Cancer Research Center (DKFZ), Department Clinical Epidemiology and Aging Reserach. Download Page for COSIMO Source Code. 2023. Available at:https://www.dkfz.de/en/klinepi/download/index.html [Accessed June 1, 2023].

[5] Brenner H, Altenhofen L, Stock C, Hoffmeister M. Prevention, early detection, and overdiagnosis of colorectal cancer within 10 years of screening colonoscopy in Germany. Clin Gastroenterol and Hepatol. 2015;13:717-23.

[6] Brenner H, Altenhofen L, Stock C, Hoffmeister M. Expected long-term impact of the German screening colonoscopy programme on colorectal cancer prevention: analyses based on 4,407,971 screening colonoscopies. Eur J Cancer. 2015;51:1346-53.

[7] Brenner H, Kretschmann J, Stock C, Hoffmeister M. Expected long-term impact of screening endoscopy on colorectal cancer incidence: A modelling study. Oncotarget. 2016;7:48168-79.

[8] Chen C, Stock C, Hoffmeister M, Brenner H. How long does it take until the effects of endoscopic screening on colorectal cancer mortality are fully disclosed?: a Markov model study. Int J Cancer. 2018;143:2718-24.

[9] Chen C, Stock C, Hoffmeister M, Brenner H. Optimal age for screening colonoscopy: a modeling study. Gastrointest Endosc. 2019;89:1017-25.e12.

[10] Dekker E, Tanis PJ, Vleugels JL, Kasi PM, Wallace MB. Colorectal cancer. Lancet. 2019;394:1467-80.

[11] Winawer SJ. Natural history of colorectal cancer. Am J Med. 1999;106:3-6.

[12] Van Rijn JC, Reitsma JB, Stoker J, Bossuyt PM, Van Deventer SJ, Dekker E. Polyp miss rate determined by tandem colonoscopy: a systematic review. Am J Gastroenterol 2006;101:343-50.

[13] Zhao S, Wang S, Pan P, Xia T, Chang X, Yang X, et al. Magnitude, risk factors, and factors associated with adenoma miss rate of tandem colonoscopy: a systematic review and meta-analysis. Gastroenterology. 2019;156:1661-74. e11.

[14] Brenner H, Zwink N, Ludwig L, Hoffmeister M. Should Screening Colonoscopy Be Offered From Age 50?: Results From a Statewide Pilot Project, and From a Randomized Intervention Study. Dtsch Ärztebl Int. 2017;114:94-100.
[15] Brenner H, Altenhofen L, Katalinic A, Lansdorp-Vogelaar I, Hoffmeister M. Sojourn time of preclinical colorectal cancer by sex and age: estimates from the German National Screening Colonoscopy Database. Am J Epidemiol. 2011;174:1140-1146.
[16] Brenner H, Altenhofen L, Stock C, Hoffmeister M. Natural history of colorectal adenomas: birth cohort analysis among 3.6 million participants of screening colonoscopy. Cancer Epidemiol Biomarkers Prev. 2013;22:1043-51.
[17] Brenner H, Altenhofen L, Stock C, Hoffmeister M. Incidence of colorectal adenomas: birth cohort analysis among 4.3 million participants of screening colonoscopy. Cancer Epidemiol, Biomarkers Prev. 2014;23:1920-7.
[18] Zentrum für Krebsregisterdaten. Krebsstatistiken für Deutschland - Interaktive Datenbank (Centre for Cancer Registry Data: Cancer Statistics for Germany - Interactive Database). 2021. Available at: ttps://www.krebsdaten.de/Krebs/DE/Datenbankabfrage/datenbankabfrage_stufe1_node.html [Accessed June 7, 2023].
[19] Weigl K, Jansen L, Chang-Claude J, Knebel P, Hoffmeister M, Brenner H. Family history and the risk of colorectal cancer: the importance of patients' history of colonoscopy. Int J Cancer. 2016;139:2213-20.
[20] Statistisches Bundesamt (Federal Office of Statistics). Allgemeine Sterbetafel 2010/2012. (General Life Table 2010/2012). 2015. Available at: https://www-genesis.destatis.de [Accessed June 7, 2023].
[21] Heisser T, Hoffmeister M, Brenner H. Effects of screening for colorectal cancer: development, documentation and validation of a multistate Markov model. Int J Cancer. 2021;148:1973-81.
[22] Heisser T, Hoffmeister M, Brenner H. Model based evaluation of long-term efficacy of existing and alternative colorectal cancer screening offers: A case study for Germany. Int J Cancer. 2022;150:1471-80.
